# Metabolomic Profiles of Essential Oils from Selected *Rosa* Varieties and Their Antimicrobial Activities

**DOI:** 10.3390/plants10081721

**Published:** 2021-08-20

**Authors:** Esraa A. Elhawary, Nada M. Mostafa, Rola M. Labib, Abdel Nasser Singab

**Affiliations:** Department of Pharmacognosy, Faculty of Pharmacy, Ain-Shams University, Cairo 11566, Egypt; esraa.elhawary@pharma.asu.edu.eg (E.A.E.); rolamilad@pharma.asu.edu.eg (R.M.L.)

**Keywords:** *Rosa* species, aspergillosis, essential oil, chemometrics, clustered heat map

## Abstract

This study aimed to analyze the essential oils of the aerial parts (A) and flowers (F) of *Rosa banksiae* var. *banksiae* Ait. (RBW), *Rosa polyantha* Thunb. “orange fairy” (RPO) and *Rosa polyantha* Thunb. “white fairy” (RPW), family Rosaceae, and perform multivariate data analyses and antimicrobial activity evaluations. The essential oil analyses were performed by GC/FID and GC/MS. Principal component analysis (PCA), hierarchical cluster analysis (HCA), and clustered heat map were used for the multivariate analyses. The antimicrobial activity was evaluated by the well-diffusion method against four bacteria and four fungi. Two hundred fifty-three compounds were identified from the six oil samples. The major components in RBW-A, RPO-A, and RPW-A were *n*-undecane (14.40, 19.36, and 9.21%) *n*-dodecane (14.54, 22.13, and 8.39%), and yomogi alcohol (8.41, 10.53, and 6.28%), respectively, whereas RBW-F, RPO-F and RPW-F contained *n*-heptadecane (16.70%), *n*-undecane (7.98%), and *β*-phellandrene (22.78%), respectively. The tested essential oils showed moderate antifungal activity against *Aspergillus fumigatus* compared to amphotericin B. PCA and HCA revealed five main clusters. The six samples carried close chemical profiles and can be regarded as fruitful sources of safe antifungal agents.

## 1. Introduction

Family Rosaceae is composed of 100 genera and 3400 species of herbs, shrubs, and trees, whereas the genus *Rosa* comprises over 250 species [1]. Traditionally, *Rosa banksiae* var. *banksiae* Ait. was used for its antifungal activity, while *Rosa polyantha* Thunb. leaves were used as a poultice applied to sores and for skincare. The ability of *Rosa* species to control microbes may be attributed to their high content of vitamin C, in hips and flowers, and other different phenolic and flavonoid constituents [2]. Indigenous people in North America traditionally used roses for cough treatment, especially in children as a decoction and inhalation therapy [3], while ancient Chinese utilized *Rosa* as a healing plant for sore throat and common cold due to its immune-stimulatory activity [4].

*Rosa banksiae* var. *banksiae* Ait. (RBW), known as White Lady Banks or Bankasian Rose, is widely cultivated in China [5]. Its leaves and flowers were reported to have antioxidant, analgesic, and wound-healing properties [6]. *Rosa polyantha* Thunb. (syn. *Rosa multiflora* Thunb.) [7] from the Greek (poly = many and anthos = flowers) usually blooms heavily and its roses come in many colors as white, orange, pink, red, yellow, and gold [8]. Several studies have reported the antioxidant and antibacterial activities of *R. polyantha* phenolic-rich extract [9,10]. The presence of drug-resistant pathogens is considered one of the important threats that may be faced in the treatment of diseases. Drug-resistant bacteria and fungi usually arise due to antibiotic misuse, poor hygienic practices, and bacterial and fungal mutations that lead to poor response to chemically based antimicrobial agents [11]. Bacteria such as *Staphylococcus aureus*, *Bacillus subtilis*, *Escherichia coli*, and *Salmonella* sp. are considered as common causes of many skin, lung, gastrointestinal, and urinary tract infections [12], while *Aspergillus fumigatus* is a saprophytic fungal strain that naturally inhabits the soil, spreads in the form of conidia, and may readily cause a serious infection called Aspergillosis in immunocompromised persons [13]. Essential oils play an important role as safe, efficient, and economic antimicrobial agents [14]. Essential oils rich in oxygenated terpenoids such as citronellol, geraniol, nerol, and thymol were reported to kill *Aspergillus* fungus through absorption into its body in the gaseous phase [13].

Recently, chemometrics has come into widespread use as an analytical tool for data discrimination [15]. Principal component analysis (PCA) and hierarchical cluster analysis (HCA) are two powerful tools for unsupervised data analysis without previous knowledge of the samples. PCA is used to discriminate large datasets while keeping their maximum variation just by clustering them according to related variables in between them [16], while HCA provides means to differentiate different groups into close clusters and sub-clusters according to their similarity level [17]. The application of chemometrics to plant metabolomics discrimination has gained much interest in recent years as a way to statistically cluster closely related samples according to their relative components, providing easy and efficient bioanalytical data analysis and representation [18].

This study aimed to identify the volatile profile obtained from the aerial parts and flowers of the most commonly cultivated *Rosa* species, belonging to family Rosaceae. Inparticular, *Rosa banksiae* var. *banksiae* Ait. (RBW), *Rosa polyantha* Thunb. “orange fairy” (RPO), and *Rosa polyantha* Thunb. “white fairy” (RPW), using GC/FID and GC/MS analyses coupled to multivariate data analysis, *viz.* PCA, HCA, and clustered heat map for differentiating the related species and varieties together with the evaluation of their antimicrobial activities. It should be noted that *Rosa polyantha* volatiles were evaluated herein for the first time.

## 2. Results and Discussion

### 2.1. Identification of the Essential Oil Components

The average yields of the hydro-distilled essential oils of RBW, RPW, and RPO essential oils, in terms of dry weight, were 0.25 ± 0.01, 0.47 ± 0.02, and 0.39 ± 0.01 mL/100 g dry weight for the aerial parts and 0.26 ± 0.02, 0.29 ± 0.02, and 0.25 ± 0.01 mL/100 g dry weight for the flowers, respectively (these values are expressed as mean ± SD of triplicate measurement). The essential oils were pale yellow in color and lighter than water. A total of 253 compounds were identified, as shown in Table S1; their GC-chromatograms are presented in Figures S1–S3 (see supplementary material Table S1, Figures S1–S3). Fatty acid-derived volatiles represented the major class in all samples, followed by the oxygenated monoterpenes. This was following all other reported *Rosa* species essential oils [19,20,21,22,23] as *R. damascena* and *R. canina* where hydrocarbons were the dominant class. For a simpler representation, the most abundant compounds (26) are compiled in Table 1. 

Comparing the analyzed data with those reported, the essential oil of *R. banksiae* Ait. flowers cultivated in China was analyzed by [6] and only 46 compounds were identified. In particular, the main constituents were dodecane (41.01%), 2-bornanone (26.34%), phenyl ethyl alcohol (5.78%), octane (4.73%), borneol (3.78%), elemicin (3.01%), *cis*-verbenol (2.68%), *α*-cadinol (1.06%), and 6-methyl dodecane (1.00%). In contrast, 71 compounds were identified in this study of *R. banksiae* cultivated in Egypt with full quantitation of each component and different essential oil compositions.

Quantities of monoterpene hydrocarbon in all groups were very small except in RPW-F. Oxygenated sesquiterpenes were average in all samples, reaching 12% except in RBW-A, while phenylpropanoids/aromatics were present in the smallest quantities in all samples together with carotenoids and sesquiterpene hydrocarbons.

Fatty acid-derived volatiles were represented by relatively high quantities of *n*-dodecane and *n*-undecane in all aerial part samples together with laurenan-2-one, while in the flower samples they were represented as (*Z*)-3-Heptenyl acetate, *n*-heptadecane, *n*-hexadecanol, *n*-nonadecane, *n*-heneicosane, and *n*-tricosane in addition to *n*-dodecane and *n*-undecane.

Monoterpene hydrocarbons were represented by *β*-phellandrene (22.78%), *α*-phellandrene (6.61%), and artemisia triene (6.21%) and were predominant in RPW-F. Nevertheless, yomogi alcohol, representing the oxygenated monoterpenes, predominated in all the tested samples except RPW-F. Alpha-bisabolol (oxygenated sesquiterpene) was only present in the RBW-F samples (3.30%).

The dominant compounds in the essential oil of RBW-A were *n*-dodecane (14.54%), *n*-undecane (14.40%), and yomogi alcohol (8.41%). Meanwhile, the following represented the major compounds in RBW-F: *n*-heptadecane (16.70%), *n*-hexadecanol (12.06%), *n*-nonadecane (11.55%), and *n*-heneicosane (8.00%).

Among the chief components isolated and identified from the RPO-A essential oil were *n*-dodecane (22.13%), *n*-undecane (19.36%), and yomogi alcohol (10.53%). The major compounds in RPO-F were *n*-undecane (7.98%), *n*-dodecane (7.96%), *n*-nonadecane (5.78%), *n*-hexadecanol (5.44%) and *n*-heneicosane (5.18%).

RPW-A showed unique composition; the essential oil was rich in *n*-undecane (9.21%), *n*-dodecane (8.39%), and yomogi alcohol (6.28%), while the flowers of RPW-F essential oil were rich in *β*-phellandrene (22.78%), *α*-phellandrene (6.61%), and artemisia triene (6.21%). 

The chemical composition of the studied essential oils showed an abundance of hydrocarbons, which was in accordance with reports on most other tested *Rosa* species essential oils [19] such as *R. damascena* [24] and *R. canina*, where hydrocarbons were the dominant class, while other studies addressed the abundance of oxygenated hydrocarbons *viz.* citronellol in *R. damascena* petals [25] and citronellol and geraniol in *R. damascena* and *R. alba* [26]. The composition pattern for any essential oil is influenced by many factors, which may be related to the plant, *viz.* genetics, plant part, sampling season, growth stage, and plant physiology or related to the environment such as climate, cultivation conditions, soil, temperature, humidity and many other factors. This may explain the variation discussed above between the three varieties and between the essential oils from the aerial parts and flowers of the same variety [27]. It is worth noting that polyantha rose essential oil content has not been reported before in the literature and, to the best of our knowledge, this is the first study comparing those three chosen *Rosa* samples’ essential oils in two different organs.

### 2.2. Screening for Antimicrobial Activity

All essential oil samples showed weak activity against *S. aureus* (RCMB010010) compared to ampicillin, with an inhibition zone of *ca.* 10.2 mm, while RPW-F showed no activity against the aforementioned bacteria. On the other hand, other tested essential oils showed no activity against *B. subtilis* (RCMB 010067). Nevertheless, *Salmonella* sp. (RCMB 010043) and *E. coli* (RCMB 010052) showed resistance against all tested essential oils except RBW-A and RPO-F with *ca*. 8 mm inhibition zone relative to the standard used. The essential oils obtained from the aerial parts and flowers of RBW, RPO, and RPW showed moderate activity against *A. fumigates* (RCMB 002008) compared to amphotericin B, while other tested fungal strains showed resistance to all samples (Table 2). As noted above, the fatty acid-derived volatile content dominated all isolated essential oils, which might explain their weak antibacterial activity. Differences in climate, harvest time, and cultivation conditions led to variable essential oil content [28,29,30], which resulted in varying degrees of antimicrobial activity.

### 2.3. Multivariate Data Analysis Using Principal Component Analysis (PCA), Hierarchical Cluster Analysis (HCA), and Clustered Heat Map

**Figure 1:**
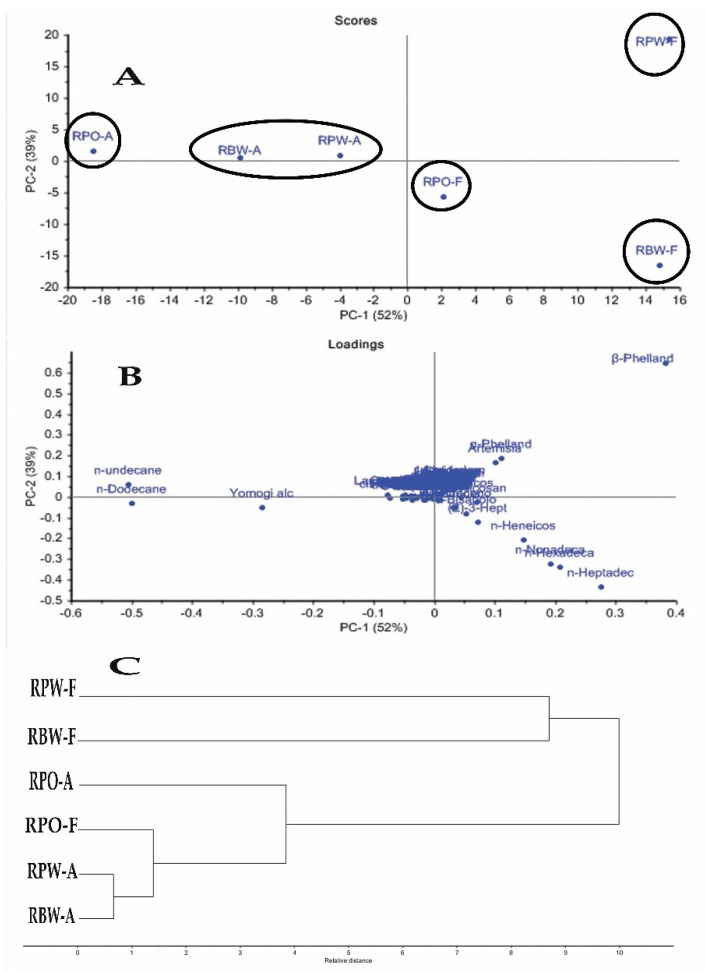
(**A**) Score plot of PC1 versus PC2 of the volatiles obtained from aerial parts and flowers of different *Rosa* varieties’ essential oils analyzed by GC/MS (*n* = 6) (area % as a variable). (**B**) Loading plot for PC1 and PC2 contributing volatiles and their assignments (area % as variable). (**C**) Dendrogram illustrates the clustering of different aerial part and flower essential oils of different *Rosa* varieties (area %). [RBW (*Rosa banksiae* var. *banksiae* Ait.), RPO (*Rosa polyantha* Thunb. “orange fairy”), RPW (*Rosa polyantha* Thunb. “white fairy”), A (aerial parts), F (Flowers)].

PCA and HCA were evaluated for the area percentage of the identified compounds. PCA analysis gave rise to five different clusters (Figure 1A,B) where the variance was 52% for PC1 and 39% for PC2. RBW-A and RPW-A were located in one cluster in the left upper quadrant, while the aerial parts of RPO were located separately in another cluster in the same quadrant. The three flower samples appeared separately in three different clusters: RBW-F and RPO-F in the right lower quadrant and RPW-F to the right in the upper quadrant. Taking into consideration that the RPO-F cluster was somewhat near the RBW-A and RPW-A clusters, the presence of a considerably rich quantity of *n*-undecane as a common component in RPO-A led to its positioning in a single cluster, yet near to the aforementioned cluster. The previously described pattern may be attributed to differences in composition between the samples where the unique abundance of *n*-dodecane and *n*-undecane implied an effect in the clustering of RBW-A and RPW-A together. Additionally, the unique presence of *n*-heptadecane in RBW-F and *β*-phellandrene in RPW-F had a powerful discriminating effect for their positioning, each in a single cluster, away from the other essential oil samples.

Concerning HCA, (Figure 1C) using area percentage revealed the closeness of composition between RBW-A and RPW-A, which were in line with PCA score plot values.

**Figure 2:**
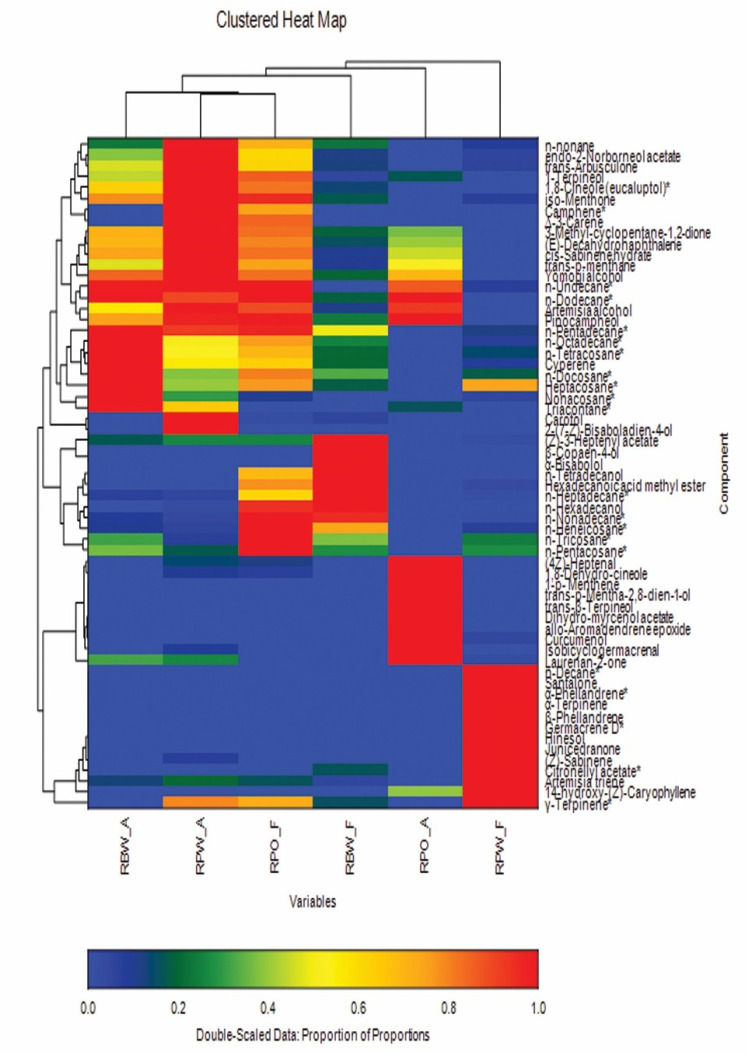
Clustered heat map showing the volatile components of the six studied *Rosa* samples. A heat map was constructed using Euclidean distance and the unweighted group method. Compounds with % composition of at least 1% were included. [RBW (*Rosa banksiae* var. *banksiae* Ait.), RPO (*Rosa polyantha* Thunb. “orange fairy”), RPW (*Rosa polyantha* Thunb. “white fairy”), A (aerial parts), F (Flowers)].

Applying further multivariate analysis with the help of clustered heat map (double dendrogram) where only compounds with area percentage ≥1% were included, the color pattern ranged from blue for the lowest area percentage and the color intensity increased gradually until red for the highest area percentage. The clustered heat map (Figure 2) confirmed the clustering results discussed above for PCA and HCA using area percentage as a variable. It was clear that the components with the highest percentage (denoted with red in the clustered heat map) in RBW-A and RPW-A were almost the same and accounted for their grouping together. 

On the other hand, based on the variation in composition, the essential oils of RPO-A, RBW-F, RPO-F, and RPW-F generated a particular arrangement in the heat map by following the pattern obtained from PCA and HCA relying on area percentage.

Plants can be a source of many bioactive metabolites [29], the profiling of which may also help in their discrimination from other inter-related species. Variation of rose essential oil chemical composition was previously studied in Tunisia for flowers of *R. canina* L. and *R. sempervirens* L., and this variability may be attributed to different cultivation localities [28]. PCA was previously applied as a chemometric tool to discriminate between different *R. damascena* Mill. genotypes [31]. PCA showed no clear clustering for different *R. alba* L. genotypes [32,33]. Multivariate analysis was used for fingerprinting of 12 *R. multiflora* genotypes, as described by [34].

## 3. Materials and Methods

### 3.1. Plant Material

Aerial parts (500 g) and flowers (50 g) of *Rosa banksiae* var. *banksiae* Ait. (syn. *Rosa banksiae* R.Br.) (RBW), known as Banksian rose, were collected from Merryland Botanical Garden, Cairo, Egypt (30°05′37′′ N, 31°18′51′′ E), while *Rosa polyantha* Thunb. “orange fairy” (syn. *Rosa multiflora* Thunb.) (RPO) and *Rosa polyantha* Thunb. “white fairy” (syn. *Rosa multiflora* Thunb.) (RPW) were collected from a private garden, Al-Mariouteya Road, Kirdassa, Giza, Egypt (30°01’13′′ N, 31°04′42′′ E). The samples were collected during March–April 2016 (flowering season) and were authenticated by agricultural engineer Terease Labib, Consultant of Plant Taxonomy at the Ministry of Agriculture, El-Orman Botanical Garden, and National Gene Bank, Giza, Egypt, and Mahmoud Abo El Nile, botanical consultant, Merryland Botanical Garden, Cairo, Egypt. Voucher specimens were kept under codes: (PHG-P-RB 165), (PHG-P-RP 205), and (PHG-P-RP 204) for RBW, RPO, and RPW, respectively, at the Herbarium of Pharmacognosy Department, Faculty of Pharmacy, Ain Shams University, Cairo, Egypt.

### 3.2. Isolation of Essential Oil Samples

Fresh aerial parts (500 g) and flowers (50 g) from each of the chosen samples were collected and cut into small pieces, then hydro-distilled using Clevenger-type apparatus, according to the Egyptian pharmacopeia [35], by boiling for 4 h. The essential oils were then collected and stored in sealed vials in the freezer at −4 °C until needed. The hydrodistillation was performed thrice. The collected essential oil samples were treated with anhydrous sodium sulfate to remove excess moisture.

### 3.3. Gas Chromatography Analysis

#### 3.3.1. GC/FID Analysis

Essential oil GC/FID analysis was performed using a method described by [30,36,37] as follows: GC analysis was accomplished using a GC HP 5890 Hewlett Packard equipped with FID and RTX-5 fused silica capillary column (30 m × 0.25 mm i.d., film thickness 0.25 μm). Sample volume: 0.2 μL of diluted sample (1:10 hexane, *v*/*v*). The oven temperature was programmed from 45 °C to 300 °C at 3 °C/min; injector temperature, 300 °C; detector temperature, 280 °C; carrier gas, helium (1.0 mL/min); automatic sample injection, 0.2 μL of diluted sample (1:10 hexane, *v*/*v*) of the essential oil; split: 1/70. The oven temperature was programmed from 45 °C to 300 °C at 3 °C/min; injector temperature, 300 °C; carrier gas, helium (0.5 mL/min); automatic sample injection, 0.02 μL of the essential oil; split: 1/70. The MS operating parameters were: interface temperature: 300 °C, ion source temperature: 200 °C, EI mode: 70 eV, scan range: 45–500 amu.

#### 3.3.2. GC/MS Analysis

Essential oils (0.2 μL of diluted samples (1:10 hexane, *v*/*v*)) were injected. GC/MS analysis was performed on a PerkinElmer quadrupole MS system (Model 5) coupled with the GC HP 5972, equipped with an RTX-5 capillary column. The resulting compounds were identified and defined according to their corresponding mass spectral fragmentation and Kovats’ indices when compared to available literature, references as Adams [38], computer library (NIST-11 Mass Spectral Library), and research articles [39,40]. The components were identified by matching their mass spectra value to the NIST library, which was then confirmed by comparing with the Kovats’ index on the RTX-5 column. Kovats’ indices were calculated from a series of hydrocarbon *n*-alkanes [C_8_–C_28_].

The following formula was used for RI calculation:

$$\mathrm{RI}=100\;[n+\left(N-n\right)x\;\left[\frac{\log\mathrm{Rt}\;\left(\mathrm{sample}\right)-(\log\mathrm{Rt}\;\left(n\right)/\left(N\right)}{(\log\mathrm{Rt}\;\left(N\right)-(\log\mathrm{Rt}\;\left(n\right)}\right]$$

where: 

RI = retention time of the identified compound.

N = no. of carbon atoms in the larger alkane.

n = no. of carbon atoms in the smaller alkane. 

### 3.4. Antimicrobial Screening

#### 3.4.1. Microorganisms

The following microbial strains were used in the screening, *viz.* Gram-positive bacterial strains as *Staphylococcus aureus* (RCMB010010) and *Bacillus subtilis* (RCMB 010067); Gram-negative bacterial strains as *Salmonella* sp. (RCMB 010043) and *Escherichia coli* (RCMB 010052); four fungi as *Aspergillus fumigatus* (RCMB 002008), *Candida albicans* (RCMB 05036), *Syncephalastrum racemosum* (RCMB 016001), and *Penicillium aurantiogriseum* (RCMB 001002). They were purchased from the Regional Center for Mycology and Biotechnology (RCMB), Cairo, Egypt. All strains were maintained in a viable state.

#### 3.4.2. Well-Diffusion Method

Screening tests regarding the inhibition zones were carried out by the agar well- diffusion method according to National Committee for Clinical Laboratory Standards [41,42]. Colonies grown overnight on an agar plate were used for inoculum suspension preparation and inoculated into Mueller-Hinton broth and malt broth for bacterial and fungal strains, respectively. Each sample of essential oils was tested at a 5 mg/mL concentration. The essential oil samples were dissolved in dimethylsulfoxide (DMSO), and 100 µL was added to each well, which was 6 mm in diameter. Then, inhibition zones were measured around each well after incubation for 24 h at 37 °C for bacteria and 28 °C for fungi. Controls using DMSO were adequately prepared. Results were compared with reference drugs, *viz.* amphotericin B, gentamicin (Sigma Chemical Co., St. Louis, MO, USA), and ampicillin G (Oxoid, UK).

### 3.5. Multivariate Data Analysis

Techniques used were unsupervised principal component analysis (PCA) and hierarchical cluster analysis (HCA), using Unscrambler 9.7 (CAMO SA, Oslo, Norway) for PCA and Hierarchical Clustering Explorer 3.5 (Human-computer interaction laboratory, University of Maryland, College Park, MD, USA) for HCA. A clustered heat map was constructed using NCSS. 12 software with Euclidean distance and the unweighted pair group method.

## 4. Conclusions

The metabolomic profiles of the essential oils (aerial parts and flowers) obtained from RBW, RPW, and RPO family Rosaceae were evaluated through GC/FID and GC/MS analyses, and a total of 253 compounds were identified, both qualitatively and quantitatively. Hydrocarbon terpenoids were the predominant class identified in the six samples except for RPWF, where oxygenated terpenoids were the most abundant. Moderate antifungal activity was traced for all the samples against *Aspergillus fumigatus* but weak activity or no activity was observed against some Gram-positive and Gram-negative bacteria. PCA and HCA results showed that RBW-A and RPW-A were clustered together due to their very close chemical composition compared to the other studied essential oil samples, and this was further confirmed by the clustered heat map.

## Figures and Tables

**Table 1:** Volatile constituents identified from the essential oils of the aerial parts and flowers of the three *Rosa* varieties (the most abundant components ≥2%).

Sr. No.	Component	KI		Peak Area %					
		Obsd.	Lit.	RBWA	RPOA	RPWA	RBWF	RPOF	RPWF
1.	Artemisia triene ^b^	925	924	0.52	-	0.51	0.23	0.35	**6.21**
2.	Yomogi alcohol ^c^	999	999	**8.41**	**10.53 **	**6.28**	2.24	**4.53**	-
3.	*α*-Phellandrene *^,b^	1005	1005	-	-	-	-	-	**6.61**
4.	*β*-Phellandrene ^b^	1032	1033	-	-	-	-	-	**22.78**
5.	*(E)*-Decahydro-Naphthalene ^h^	1054	1054	**2.24**	**2.14**	1.95	0.81	1.38	0.43
6.	*cis*-Sabinene hydrate ^c^	1063	1068	**2.76**	**2.7**	**2.29**	0.69	1.67	0.42
7.	(*Z*)-3-Heptenyl acetate ^a^	1095	1098	0.73	-	0.71	**4.92**	0.61	0.13
8.	*n*-Undecane *^,h^	1100	1100	**14.4**	**19.36**	**9.21**	0.94	**7.98**	**2.88**
9.	*iso*-Menthone ^c^	1163	1164	**2.43**	-	1.96	0.62	1.62	0.34
10.	*n*-Dodecane *^,h^	1199	1200	**14.54**	**22.13**	**8.39 **	**4.77**	**7.96**	**2.75**
11.	Carotol ^e^	1594	1594	-	-	**2.35**	0.23	0.04	-
12.	*allo*-Aromadendrene epoxide ^e^	1637	1646	-	**2.51**	-	-	-	-
13.	14-Hydroxy-(*Z*)-Caryophyllene ^e^	1667	1667	-	0.81	-	-	-	**2.12**
14.	*α*-Bisabolol ^e^	1681	1683	-	-	-	**3.30**	-	-
15.	*n*-Heptadecane *^,h^	1700	1700	0.99	-	0.47	**16.70**	**4.88**	0.33
16.	*n*-Hexadecanol ^h^	1875	1881	-	-	0.17	**12.06**	**5.44**	-
17.	Curcumenol ^e^	1730	1734	-	**2.43**	-	-	-	0.12
18.	Isobicyclo-Germacrenal ^e^	1732	1741	-	**2.30**	0.08	-	-	-
19.	*n*-Nonadecane *^,h^	1900	1900	0.91	-	0.29	**11.55**	**5.78**	0.2
20.	Hexadecanoic acidmethyl ester ^a^	1920	1916	-	-	-	**2.04**	0.76	0.09
21.	*n*-Heneicosane *^,h^	2101	2100	0.91	-	0.33	**8.00**	**5.18**	1.04
22.	Laurenan-2-one ^h^	2120	2116	0.71	**3.40**	0.37	-	-	0.08
23.	*n*-Tricosane *^,h^	2301	2300	1.98	-	0.28	**2.71**	**3.45**	**2.40**
24.	*n*-Pentacosane *^,h^	2500	2500	1.80	-	0.54	1.56	**2.70**	**2.11**
25.	Heptacosane *^,h^	2700	2700	**2.02**	-	0.51	0.42	0.85	**2.35**
26.	Nonacosane *^,h^	2885	2900	**1.99**	-	0.38	-	0.11	0.16
**Total identified (%)**				**89.61**	**94.83**	**85.76**	**93.55**	**92.54**	**94.66**

RBW (*Rosa banksiae* var. *banksiae*), RPO (*Rosa polyantha* orange fairy), RPW (*Rosa polyantha* white fairy), A (Aerial parts) and F (Flowers). (*) is for components reported before for different *Rosa* species volatile oil; bolded numbers for components with concentrations ≥2%. a: fatty acid-derived volatiles, b: monoterpene hydrocarbons, c: oxygenated monoterpenes, e: oxygenated sesquiterpenes, h: miscellaneous.

**Table 2:** Antimicrobial activity of aerial parts and flower volatile oils of *Rosa* varieties expressed as the diameter of one of inhibition in mm.

Tested Microorganisms	RBW-A	RPO-A	RPW-A	RBW-F	RPO-F	RPW-F	Control
**Gram-Positive Bacteria**							Ampicillin
*Staphylococcus aureus* (RCMB010010)	11	8	8	11	13	NA	23
*Bacillus subtilis* (RCMB 010067)	8	NA	NA	NA	8	NA	32
**Gram-Negative Bacteria**							Gentamicin
*Salmonella* sp. (RCMB 010043)	NA	NA	NA	NA	NA	NA	17
*Escherichia coli* (RCMB 010052)	NA	NA	NA	NA	NA	NA	19
**Fungi**							Amphotericin B
*Aspergillus fumigatus* (RCMB 002008)	14	15	11	14	15	12	23
*Candida albicans* (RCMB 05036)	NA	NA	NA	NA	NA	NA	25
*Syncephalastrum racemosum* (RCMB 016001)	NA	NA	NA	NA	NA	NA	20
*Penicillium aurantiogriseum* (RCMB 001002)	NA	NA	NA	NA	NA	NA	21

RBW (*Rosa banksiae* var. *banksiae* Ait.), RPO (*Rosa polyantha* Thunb. “orange fairy”), RPW (*Rosa polyantha* Thunb. “white fairy”), A (aerial parts), F (Flowers), NA (not active).

## Data Availability

Data are available upon request from the authors.

## Supplementary Materials

The following are available online at https://www.mdpi.com/article/10.3390/plants10081721/s1, Table S1. Volatile constituents identified in the volatile oils of the aerial parts and flower of different *Rosa* varieties, Figure S1. GC-chromatogram of the essential oil of *Rosa banksiae* var. *banksiae* Ait. (**A**) Aerial parts (RBW-A), (**B**) Flowers (RBW-F), Figure S2. GC-chromatogram of the essential oil of Rosa polyantha Thunb. ‘orange fairy’ (A) Aerial parts (RPO-A), (B) Flowers (RPO-F), Figure S3. GC-chromatogram of the essential oil of *Rosa polyantha* Thunb. ‘white fairy’ (**A**) Aerial parts (RPW-A), (**B**) Flowers (RPW-F).
