# Supplementary material for: Metabolomic Profiles of Essential Oils from Selected Rosa Varieties and Their Antimicrobial Activities
Source: Plants (Basel). 2021 Aug 20;10(8):1721. doi: 10.3390/plants10081721 (PMC8398089; doi:10.3390/plants10081721)
Supplement: Supplementary file 1 [file plants-10-01721-s001.zip › plants-1332659-supplementary.pdf]

Supplementary material

Article

Metabolomic Profiles of Essential Oils from Selected *Rosa* Varieties and their Antimicrobial Activities

Esraa A. Elhawary[#], Nada M. Mostafa[#,*], Rola M. Labib, and Abdel Nasser Singab[*]

Department of Pharmacognosy, Faculty of Pharmacy, Ain-Shams University, Cairo 11566, Egypt; esraa.elhawary@pharma.asu.edu.eg (E.A.E.); nadamostafa@pharma.asu.edu.eg (N.M.M.); rolamilad@pharma.asu.edu.eg (R.M.L.); dean@pharma.asu.edu.eg (A.N.S.)

# These authors contributed equally to this work

* Correspondence: A.N.S., dean@pharma.asu.edu.eg; Tel.: +20224051120; N.M.M., nadamostafa@pharma.asu.edu.eg; Tel.: +201025666872

Abstract: This study aimed to analyze the essential oils of aerial parts (A) and flowers (F) of *Rosa banksiae* var. *banksiae* Ait. (RBW), *Rosa polyantha* Thunb. 'white fairy' (RPW) and *Rosa polyantha* Thunb. 'orange fairy', family Rosaceae, together with multivariate data analyses and antimicrobial activity evaluation. The essential oils analyses were performed by GC/FID and GC/MS. Principal Component Analysis (PCA), Hierarchical Cluster Analysis (HCA) and clustered heatmap were used for the multivariate analyses. The antimicrobial activity was evaluated by well-diffusion method against four bacteria and four fungi. Two hundred fifty-three compounds were identified from the six oil samples. The major components in RBW-A, RPO-A, and RPW-A were *n*-undecane (14.40, 19.36, and 9.21 %) *n*-dodecane (14.54, 22.13, and 8.39 %), and yomogi alcohol (8.41, 10.53, and 6.28 %), respectively. While in RBW-F, RPO-F and RPW-F were *n*-heptadecane (16.70 %), *n*-undecane (7.98 %), and *β*-phellandrene (22.78 %), respectively. The tested essential oils showed moderate antifungal activity against *Aspergillus fumigatus* compared to amphotericin B. PCA, HCA revealed five main clusters and the clustered heatmap showed the highest concentrations in red and the lowest ones in blue. The six samples carry close chemical profiles and can be regarded as fruitful sources of safe antifungal agents.

Keywords: *Rosa* species; Aspergillosis; essential oil; chemometrics; clustered heatmap

Table S1. Volatile constituents identified in the aerial parts and flower the volatile oils of different *Rosa* varieties.

Sr. No.	Component	KI		Peak area %						Sr. No.	Component	KI		Peak area %					
		Obsd.	Lit.	RBW A	RPO A	RPW A	RBW F	RPO F	RPW F			Obsd.	Lit.	RBW A	RPO A	RPW A	RBW F	RPO F	RPW F
1.	*n*-Octane*[h]	800	800	0.29	-	0.33	0.08	0.19	0.08	13.	**Artemisia triene**[b]	925	924	0.52	-	0.51	0.23	0.35	**6.21**
2.	Ethyl butanoate[a]	804	804	0.19	-	0.14	0.13	0.09	0.03	14.	Ethyl tiglate[a]	931	944	0.52	-	0.60	0.21	0.38	0.13
3.	Isopropyl butanoate[a]	847	848	0.27	-	0.20	0.18	0.13	0.05	15.	Tetrahydro-Citronellene[b]	937	937	-	-	0.05	-	-	-
4.	(2*E*)-Hexenal[h]	855	853	-	-	0.27	-	0.17	0.04	16.	*β*-Citronellene[b]	940	947	-	-	0.24	-	0.06	-
5.	(*E*)-Salvene[h]	866	867	-	-	0.17	-	0.11	0.03	17.	*α*-Fenchene[b]	945	953	-	-	0.09	-	0.04	-
6.	Santene[h]	879	884	0.25	0.38	0.25	0.09	0.15	0.04	18.	3-Hepten-1-ol[h]	949	954	0.38	-	0.66	0.18	0.42	0.14
7.	*n*-Nonane[h]	890	900	0.51	-	1.39	0.57	0.86	0.27	19.	Camphene*[b]	954	955	-	-	1.17	-	0.76	-
8.	(4*Z*)-Heptenal[h]	895	904	-	1.88	0.11	-	0.08	-	20.	(*E*)-2-Hepten-1-ol[h]	956	965	0.48	-	0.43	-	0.28	0.12
9.	Santolina triene[b]	908	908	-	-	0.08	-	0.02	0.43	21.	Ethyl-3-methyl Pentanoate[a]	959	960	0.80	0.60	0.70	0.28	0.46	0.19
10.	Isocitronellene[b]	913	926	-	-	0.18	-	0.09	0.04	22.	(*E*)-Pinane[b]	962	972	-	-	0.14	-	0.10	-
11.	2,5-Diethenyl-2-methyl-Tetrahydrofuran[h]	916	914	-	-	0.35	-	0.20	0.07	23.	(*Z*)-4-Hepten-1-ol[h]	966	870	0.44	-	0.60	0.17	0.42	0.17
12.	Ethyl -2-methyl-4-Pentenoate[a]	921	926	0.59	0.56	0.76	0.22	0.46	0.12	24.	Artemiseole[c]	968	976	0.24	-	0.48	0.08	0.32	0.10
25.	1-Octen-3-one[h]	971	980	-	-	0.61	-	0.39	0.27	39.	***α*-Phellandrene***[b]	1005	1005	-	-	-	-	-	**6.61**

26.	*(Z)*-Sabinene[b]	974	975	-	-	0.06	-	-	1.96	40.	Dehydroxy-*cis*-Linalool oxide[c]	1008	1008	-	-	0.21	-	0.24	0.04
27.	exo-2-Neoborneol[h]	975	976	-	-	0.12	-	0.09	-	41.	*δ*-3-Carene[b]	1011	1011	-	-	0.15	-	0.11	-
28.	*trans*-*p*-Menthane[b]	979	979	0.92	1.53	1.12	0.36	0.75	0.25	42.	1,4-Cineole[c]	1014	1016	-	-	0.24	-	0.16	-
29.	3-*p*-Menthene[b]	983	988	0.74	-	0.88	0.26	0.59	0.16	43.	*α*-Terpinene[b]	1017	1018	-	-	-	-	-	1.16
30.	6-Methyl-5-Hepten-2-one[h]	985	986	-	-	0.25	-	0.17	-	44.	3-Methyl-Cyclopentane-1,2-dione[h]	1021	1043	1.5	1.18	1.33	0.46	0.96	-
31.	1-Decene[h]	988	993	-	-	0.16	-	0.11	-	45.	Allyl tiglate[a]	1023	1022	-	-	0.37	-	0.25	-
32.	*cis*-*meta*-Mentha-2,8-diene[b]	990	993	-	-	-	-	-	0.88	46.	*p*-Cymene*[b]	1024	1025	0.26	-	-	-	-	0.37
33.	1,8-Dehydro-Cineole[c]	991	994	-	1.54	0.06	-	0.04	-	47.	Limonene*[b]	1029	1028	-	-	-	0.15	-	-
34.	Dehydro-*trans*-Llinalool oxide[c]	993	993	-	-	0.26	-	0.19	0.04	48.	1-*p*- Menthene[b]	1030	1032	-	1.11	-	-	-	-
35.	Butyl butanoate[a]	994	1002	0.67	-	0.37	0.23	0.27	0.07	49.	1,8-Cineole (Eucaluptol)*[c]	1031	1033	1.83	-	1.85	0.45	1.32	-
36.	**Yomogi alcohol**[c]	999	999	**8.41**	**10.53**	**6.28**	2.24	**4.53**	-	50.	***β*-Phellandrene**[b]	1032	1033	-	-	-	-	-	**22.78**
37.	*n*-Decane*[h]	1000	1000	-	-	-	-	-	1.82	51.	(*E*)3-Octen-2-one[h]	1033	1034	0.46	0.48	0.57	0.15	0.41	-
38.	*p*-Mentha-7,8-diene[b]	1004	1004	-	-	0.26	-	0.23	-	52.	(*Z*)-*β*-Ocimene*[b]	1036	1040	0.29	-	0.56	-	0.38	0.11
										53.	Lavenderlactone[g]	1039	1041	0.21	-	0.29	-	0.22	0.05
54.	Propyl Tiglate[a]	1038	1038	-	-	0.45	-	0.33	0.13	67.	*trans*-Linalool oxide*[c]	1077	1073	-	-	0.25	-	0.19	-
55.	*cis*-Dihydro-Rose oxide[c]	1043	1043	-	-	-	-	-	0.03	68.	Artemisia alcohol[c]	1083	1092	0.90	2.13	0.95	0.31	0.74	0.18
56.	*cis*-Arbusculone[g]	1045	1051	-	-	0.18	-	0.14	0.12	69.	2-Nonanol[h]	1089	1097	0.51	0.44	0.64	0.17	0.51	-
57.	Dihydro-Tagetone[c]	1047	1047	-	-	0.30	-	0.23	-	70.	*p*-Mentha-2,4(8)-diene[b]	1090	1088	-	-	-	-	-	0.82
58.	(*E*)-*β*-Ocimene*[b]	1049	1049	-	-	-	-	-	0.40	71.	Linalool*[c]	1093	1098	-	-	0.19	-	0.14	0.02

59.	(*Z*)-3-Octen-1-ol[h]	1051	1051	-	-	0.52	-	0.38	0.08	72.	**(*Z*)-3-Heptenyl acetate**[a]	1095	1098	0.73	-	0.71	**4.92**	0.61	0.13
60.	*cis*-Linalool oxide*[c]	1066	1058	-	0.83	0.22	-	0.19	-	73.	***n*-Undecane***[h]	1100	1100	**14.4**	**19.36**	**9.21**	0.94	**7.98**	2.88
61.	(*E*)-Decahydro-Naphthalene[h]	1054	1054	2.24	2.14	1.95	0.81	1.38	0.43	74.	*α*-Pinene oxide[c]	1101	1103	0.47	-	-	0.69	-	-
62.	(*E*)-2-Octen-1-al[h]	1056	1062	0.78	0.52	0.73	0.26	0.54	-	75.	Maltol[h]	1105	1108	-	-	0.25	0.53	0.24	-
63.	*γ*-Terpinene*[b]	1060	1064	-	-	0.62	0.22	0.49	1.93	76.	*trans*-Vertocitral C[c]	1106	1106	-	-	-	-	-	0.41
64.	*cis*-Sabinene hydrate[c]	1063	1068	2.76	2.7	2.29	0.69	1.67	0.42	77.	*β*-Thujone[c]	1110	1114	0.90	0.63	0.94	0.29	0.73	0.22
65.	*trans*-Arbusculone[g]	1070	1070	1.15	-	1.57	0.36	0.84	0.23	78.	endo-Fenchol[c]	1117	1117	0.71	-	0.79	-	0.70	0.30
66.	*trans*-Dihydro-Rose oxide[c]	1073	1073	-	-	0.27	-	0.22	0.03	79.	*trans-p*-Mentha-2,8-dien-1-ol[c]	1126	1128	-	1.00	-	-	-	-
80.	Dehydro-Sabinaketone[c]	1120	1125	-	-	0.23	-	0.19	0.03	91.	Isobutyl hexanoate[h]	1154	1149	0.36	-	0.72	-	0.64	0.09
81.	*trans*-Pinene hydrate[c]	1123	1123	-	-	0.39	-	0.33	-	92.	*trans*-*β*-Terpineol[c]	1162	1163	-	1.88	-	-	-	-
82.	exo-Fenchol[c]	1124	1122	-	-	-	-	-	0.53	93.	Dihydro-Myrcenol acetate[c]	1212	1214	-	1.88	-	-	-	-
83.	endo-2-Norborneol acetate[c]	1127	1128	1.12	-	1.84	0.39	0.97	0.24	94.	Khusilal[g]	1648	1648	-	0.94	-	-	-	0.09
84.	exo-2-Norborneol acetate[c]	1129	1129	-	-	-	-	0.12	-	95.	*cis*-Dihydro-*β*-Terpineol[c]	1159	1160	0.75	0.61	0.77	0.23	0.68	0.13
85.	1-Terpineol[c]	1134	1134	1.32	0.92	1.78	0.33	1.34	0.24	96.	*iso*-Menthone[c]	1163	1164	2.43	-	1.96	0.62	1.62	0.34
86.	*trans*-Dihydro-*β*-Terpineol[c]	1138	1138	-	-	0.45	-	0.39	-	97.	*neo*-Menthol[c]	1165	1176	-	-	0.41	-	0.35	-

87.	*cis*-Verbenol[c]	1141	1141	-	-	0.18	-	0.15	-	98.	Pinocampheol[c]	1169	1173	0.94	1.9	0.77	0.34	0.68	-
88.	*cis*-Pinene hydrate[c]	1143	1143	-	-	0.23	-	0.21	0.46	99.	Rose furan epoxide[g]	1172	1172	-	-	0.15	-	0.17	-
89.	Camphene hydrate[c]	1149	1150	-	-	0.58	-	0.48	-	100.	*cis*-Linalool oxide (pyranoid)[c]	1174	1174	-	-	0.12	-	-	0.04
90.	Isopulegol*[c]	1152	1156	-	-	-	-	-	0.06	101.	*cis*-Pinocamphene[b]	1175	1175	-	-	-	-	0.12	-
102.	*trans*- Linalool oxide (pyranoid)[c]	1176	1179	-	-	0.20	-	0.17	-	116.	*cis*-Sabinene hydrate acetate[c]	1221	1221	-	-	0.14	-	0.11	-
103.	Santalone[h]	1180	1181	-	-	-	-	-	1.62	117.	*β*-Citronellol*[c]	1222	1228	-	-	0.13	-	0.15	-
104.	2-Methyl-*iso*-Borneol[c]	1181	1183	-	-	0.46	-	0.42	-	118.	endo-Fenchyl acetate*[c]	1226	1225	-	-	0.03	-	-	-
105.	Cryptone[c]	1185	1186	0.38	-	0.73	-	0.66	0.17	119.	*cis*-Carveol[c]	1231	1231	-	-	-	-	-	0.08
106.	*Neo*-*iso*-Verbanol[c]	1189	1189	0.47	-	0.46	0.48	0.42	0.35	120.	exo-Fenchyl acetate*[c]	1234	1232	-	-	-	-	-	0.02
107.	*cis*-Dihydro-Carvone[c]	1192	1194	-	-	0.09	-	0.11	-	121.	Carvone[c]	1239	1242	0.42	0.59	0.31	0.12	0.25	0.10
108.	*α*-Terpineol*[c]	1193	1195	-	-	-	-	-	0.53	122.	*O*-Anisaldehyde[f]	1242	1242	-	-	0.11	-	0.05	-
109.	*cis*-*p*-Menthan-2-one[c]	1196	1195	-	-	-	-	0.23	-	123.	Cuminaldehyde[f]	1244	1246	-	-	-	-	-	0.09
110.	***n*-Dodecane***[h]	1199	1200	**14.54**	**22.13**	**8.39**	**4.77**	**7.96**	2.75	124.	Piperitone[c]	1252	1252	-	-	0.08	-	0.07	0.03
111.	*trans*-Dihydro-Carvone[c]	1205	1207	-	-	-	-	0.30	-	125.	*cis*-Piperitone epoxide[c]	1254	1256	-	-	0.07	-	0.07	-
112.	*n*-Decanal[h]	1206	1204	-	-	0.27	0.37	-	0.08	126.	(*Z*)-4-Decen-1-ol[h]	1259	1257	-	-	0.06	-	0.07	-
113.	*trans*-Piperitol[c]	1209	1212	-	-	0.12	-	0.12	-	127.	(*E*)-2-Decenal[h]	1264	1264	-	-	0.13	-	0.15	0.16
114.	Linalool formate[c]	1211	1219	-	-	-	-	-	0.33	128.	*n*-Decanol[h]	1273	1275	-	-	0.04	-	0.04	-
115.	*β*-Cyclocitral*[c]	1219	1224	-	-	0.06	-	0.06	-	129.	Dihydro-Linalool	1281	1287	-	-	-	-	-	0.11

											acetate[c]								
130.	2-Ethyl-exo-Fenchol[c]	1297	1297	-	-	-	-	-	0.07	143.	Linalool butanoate[c]	1423	1423	-	-	0.10	-	-	-
131.	*n*-Tridecane*[h]	1301	1300	0.23	0.67	0.16	0.22	0.16	0.06	144.	4,8-*β*-epoxy-Caryophyllane[e]	1424	1434	-	-	-	-	0.10	-
132.	Undecanal[h]	1310	1310	-	-	-	-	-	0.10	145.	(*E*)-*α*-Ionone*[g]	1427	1427	-	-	0.16	-	0.13	0.48
133.	*cis*-Dihydro-*α*-Terpinyl acetate[c]	1322	1317	-	-	-	-	-	0.81	146.	*β*-Copaene*[d]	1436	1439	-	-	-	-	-	0.07
134.	Citronellyl acetate*[c]	1355	1356	-	-	-	0.16	-	1.34	147.	*γ*-Elemene[d]	1439	1441	-	-	-	-	-	0.25
135.	Hydroxyl Citronellol[c]	1363	1362	-	-	0.04	-	0.04	0.05	148.	*α*-Himachalene[d]	1447	1449	-	-	-	-	-	0.27
136.	Neryl acetate[c]	1366	1365	-	-	-	0.12	-	0.79	149.	Citronellyl propanoate[a]	1448	1444	-	-	-	-	0.08	-
137.	Linalool isobutanoate[c]	1375	1375	-	-	0.05	-	0.05	-	150.	*α*-epi-Cedrane[d]	1452	1449	-	-	-	-	0.04	0.13
138.	*β*-Patchoulene[d]	1381	1381	-	-	-	-	-	0.10	151.	Sesquisabinene[d]	1456	1461	-	-	-	-	-	0.06
139.	7-epi-Sesquithujene[d]	1385	1391	-	-	-	-	-	0.11	152.	Cabreuva oxide B[e]	1462	1466	-	-	0.18	-	-	0.12
140.	*α*-Chamipinene[d]	1396	1396	-	-	0.12	-	-	0.13	153.	*cis*-Cadina-1(6), 4-diene[d]	1463	1463	-	-	-	-	0.18	-
141.	Cyperene[d]	1398	1398	1.7	-	0.60	0.39	0.59	0.21	154.	9-*epi*-(*E*)-Caryophyllene[d]	1467	1465	-	-	0.03	-	-	-
142.	*α*-Thujaplicin[c]	1408	1400	-	-	0.10	-	0.10	-	155.	*cis*-Thujopsadiene[d]	1470	1468	-	-	0.05	-	0.05	0.08
156.	Amorpha-4,7(11)-diene[d]	1482	1484	-	-	-	-	-	0.07	169.	Longipinanol[e]	1567	1567	-	-	0.05	-	-	-
157.	Germacrene D*[d]	1485	1487	-	-	-	-	-	0.45	170.	Spathulenol[e]	1578	1585	-	-	0.05	-	-	0.09
158.	*trans*-Muurola-4(14),5-	1490	1494	-	-	-	-	-	0.46	171.	*α*-Cadinene[d]	1545	1535	-	-	-	-	-	0.40

	diene[d]																		
159.	*n*-Pentadecane*[h]	1500	1500	1.15	-	0.67	0.65	0.61	0.21	172.	Selina-3,7(11)-diene[d]	1551	1547	-	-	-	-	-	0.22
160.	*trans*-β-Guaiene[d]	1505	1503	-	-	-	-	-	0.35	173.	α-Cedrene epoxide[e]	1568	1575	-	-	-	-	-	0.74
161.	δ-Amorphene[d]	1508	1511	-	-	-	-	-	0.28	174.	α-Calacorene[d]	1552	1549	-	-	0.12	-	-	-
162.	Nootkatene[d]	1516	1518	-	-	-	-	-	0.05	175.	*trans*-Dauca-4(11),7-diene[d]	1558	1557	-	-	-	-	-	0.47
163.	δ-Cadinene*[d]	1523	1523	-	-	-	-	-	0.28	176.	Germacrene B[d]	1563	1561	-	-	-	-	-	0.09
164.	γ-Cuprenene[d]	1532	1533	-	-	-	-	-	0.91	177.	β-(*E*)-Ionol acetate[g]	1541	1535	-	-	-	-	-	0.04
165.	γ-Dehydro-ar-Himachalene[d]	1537	1527	-	-	0.04	-	-	-	178.	10-*epi*-Cubebol[e]	1535	1534	-	-	-	-	-	0.05
166.	*cis*-Sesqui-Sabinene hydrate[e]	1546	1559	-	-	0.07	-	-	-	179.	β-Copaen-4-ol[e]	1587	1585	-	-	-	0.21	-	-
167.	α-Agarofuran[e]	1553	1552	-	-	-	-	0.04	-	180.	Carotol[e]	1594	1594	-	-	2.35	0.23	0.04	-
										181.	Globulol[e]	1595	1588	-	-	-	-	-	0.84
168.	2-Methyl-Pentadecan[d]	1562	1564	-	-	0.12	-	0.05	-	182.	*n*-Hexadecane*[h]	1597	1600	0.95	-	0.46	0.49	0.52	0.16
183.	Guaiol[e]	1606	1602	-	-	-	-	-	0.37	197.	α-Cadinol*[e]	1654	1658	-	-	-	-	-	0.49
184.	α-Eudesmol*[e]	1619	1620	-	-	-	-	-	0.03	198.	γ-Muurolene[e]	1658	1488	-	-	-	-	-	0.48
185.	2-(7-*Z*)-Bisaboladien-4-ol[e]	1621	1619	-	-	0.16	-	-	-	199.	Junicedranone[e]	1665	1665	-	-	-	-	-	2.03
186.	1-*epi*-Cubenol*[e]	1625	1627	-	-	0.05	-	-	-	200.	7-*epi*-α-Eudesmol[e]	1666	1658	-	-	0.86	0.64	-	-
187.	*trans*-Isolongifolanone[e]	1629	1627	-	-	-	-	-	0.50	201.	*n*-Tetradecanol[h]	1678	1676	-	-	-	1.91	0.63	-
188.	γ-Eudesmol*[e]	1632	1633	-	0.64	-	-	-	0.79	202.	14-Hydroxy-(*Z*)-Caryophyllene[e]	1667	1667	-	0.81	-	-	-	2.12
189.	*allo*-Aromadendrene	1637	1646	-	2.51	-	-	-	-	203.	Elemol acetate[e]	1680	1679	-	-	-	-	0.56	0.57

	epoxide[e]																		
190.	*cis*-Cadina-4-en-7-ol[e]	1639	1636	-	-	0.09	-	-	0.11	204.	***α*-Bisabolol**[e]	1681	1683	-	-	-	**3.30**	-	-
191.	1,7-*diepi*-*α*-Cedrenal[e]	1643	1643	-	-	0.08	0.52	0.08	-	205.	Eudesma-4(15),7-dien-1-ol[e]	1683	1688	-	-	0.16	-	-	0.05
192.	Hinesol[e]	1644	1640	-	-	-	-	-	1.30	206.	*epi*-*α*-Bisabolol[e]	1684	1686	-	-	-	-	-	0.09
193.	2,6,10-Trimethyl-Pentadecane[h]	1647	1649	-	-	-	-	0.05	-	207.	5-*neo*-Cedranol[e]	1685	1686	-	-	-	0.42	-	-
194.	(*Z*)-Amyl cinnamaldehyde[f]	1649	1649	-	-	0.23	-	-	-	208.	2,3-Dihydro-Farnesol[e]	1689	1689	-	-	-	0.60	0.09	-
195.	Valerianol[e]	1652	1655	-	-	-	-	-	0.45	209.	***n*-Heptadecane***[h]	1700	1700	0.99	-	0.47	**16.70**	**4.88**	0.33
196.	Himachalol*[e]	1653	1656	-	-	0.09	-	-	-	210.	Sesquicineol-2-n[e]	1705	1702	-	-	0.17	-	-	0.07
211.	*cis*-Thujopsenal[e]	1710	1709	-	-	0.09	-	-	-	226.	Cyclo-Pentadecanolide[h]	1846	1806	-	-	0.11	-	-	0.05
212.	Mayurone[e]	1711	1710	-	-	-	-	-	0.43	227.	***n*-Hexadecanol**[h]	1875	1881	-	-	0.17	**12.06**	**5.44**	-
213.	(*Z*)-*α*-Alantone[e]	1716	1718	-	-	-	-	0.02	-	228.	(5*Z*, 9*E*)-Farnesyl acetone[g]	1880	1880	-	-	-	0.50	0.15	-
214.	*iso*-Longifolol[e]	1724	1723	-	-	-	0.43	0.12	-	229.	***n*-Nonadecane***[h]	1900	1900	0.91	-	0.29	**11.55**	**5.78**	0.22
215.	14-Hydroxy-4,5-dihydro-Caryophyllene[e]	1706	1706	-	0.76	-	-	0.44	-	230.	(5*E*, 9*E*)-Farnesyl acetone[g]	1904	1913	-	-	-	0.61	0.18	-
216.	Curcumenol[e]	1730	1734	-	2.43	-	-	-	0.12	231.	Hexadecanoic acidmethyl ester[a]	1920	1916	-	-	-	2.04	0.76	0.09
217.	Isobicyclo-Germacrenal[e]	1732	1741	-	2.30	0.08	-	-	-	232.	Nootkatin[h]	1958	1961	-	-	-	0.23	0.18	-
218.	Cyclo-Colorenone[e]	1749	1748	-	0.67	-	-	-	-	233.	*n*-Eicosane*[h]	1995	2000	0.67	-	0.24	0.64	0.62	0.14
219.	(2*Z*, 6*E*)-Farnesol*[e]	1728	1700	-	-	-	-	-	0.10	234.	(6*Z*,10*E*)-Pseudo-Phytol[h]	2022	2031	-	-	-	-	-	0.08
220.	*γ*-(*Z*)-Curcumen-12-ol[e]	1729	1729	-	-	0.23	-	-	-	235.	*n*-Octadecanol[h]	2075	2080	-	-	-	0.35	0.26	-

221.	*n*-Pentadecanol[h]	1774	1778	-	-	-	0.14	0.07	-	236.	Dehydro-Juvibie[h]	2080	2085	-	-	-	-	0.04	-
222.	Benzyl benzoate[f]	1775	1775	-	-	0.08	-	-	-										
223.	(*Z*)-*α*-Santalol acetate [e]	1779	1669	-	-	0.32	-	-	-	237.	***n*-Heneicosane*[h]**	2101	2100	0.91	-	0.33	**8.00**	**5.18**	1.04
224.	14-Hydroxy-*α*-Muurolene[e]	1782	1782	-	-	0.21	-	-	0.14	238.	Linolenic acid methyl ester[a]	2109	2108	-	-	0.49	0.30	0.08	0.34
225.	*n*-Octadecane*[h]	1796	1800	0.96	-	0.31	0.28	0.40	0.11	239.	**Laurenan-2-one**[h]	2120	2116	0.71	**3.40**	0.37	-	-	0.08
240.	*n*-Docosane*[h]	2203	2200	0.95	-	0.23	0.36	0.42	0.27	248.	*n*-Pentacosane*[h]	2500	2500	1.80	-	0.54	1.56	2.70	2.11
241.	(*E*)-Phytol acetate[a]	2232	2218	-	-	-	-	-	0.10	249.	Hexacosane*[h]	2601	2600	0.55	-	0.34	-	0.13	0.19
242.	(*Z*)-9-Tricosene*[h]	2294	2271	-	-	-	-	-	0.13	250.	Heptacosane*[h]	2700	2700	2.02	-	0.51	0.42	0.85	2.35
243.	***n*-Tricosane***[h]	2301	2300	1.98	-	0.28	2.71	**3.45**	2.40	251.	Octacosane[h]	2794	2800	-	-	0.11	-	0.03	0.03
244.	3-*α*-14,15-Dihydro-Manool oxide[h]	2331	2338	-	-	-	-	-	0.04	252.	Nonacosane*[h]	2885	2900	1.99	-	0.38	-	0.11	0.16
245.	*n*-Tetracosane*[h]	2395	2400	1.07	-	0.36	0.25	0.41	0.24	253.	Triacontane*[h]	3059	3000	1.02	0.25	0.42	-	-	-
246.	(*E*)-Labd-13-en-8,15-diol[h]	2431	2422	-	-	-	-	-	0.16										
247.	Drimenol[h]	2494	1750	-	-	-	-	-	0.11										
	**Total identified (%)**			**89.61**	**94.83**	**85.76**	**93.55**	**92.54**	**94.66**										

RBW (*Rosa banksiae* var. *banksiae*), RPO (*Rosa polyantha* orange fairy), RPW (*Rosa polyantha* white fairy), A (Aerial parts) and F (Flowers). (*) is for components reported before for different *Rosa* species volatile oil; bolded numbers for components with concentrations ≥3%

a: fatty acid-derived volatiles, b: monoterpene hydrocarbons, c: oxygenated monoterpenes, d: sesquiterpene hydrocarbons, e: oxygenated sesquiterpenes, f: phenylpropanoids/aromatics, g: carotenoid-derived volatiles, h: miscellaneous.

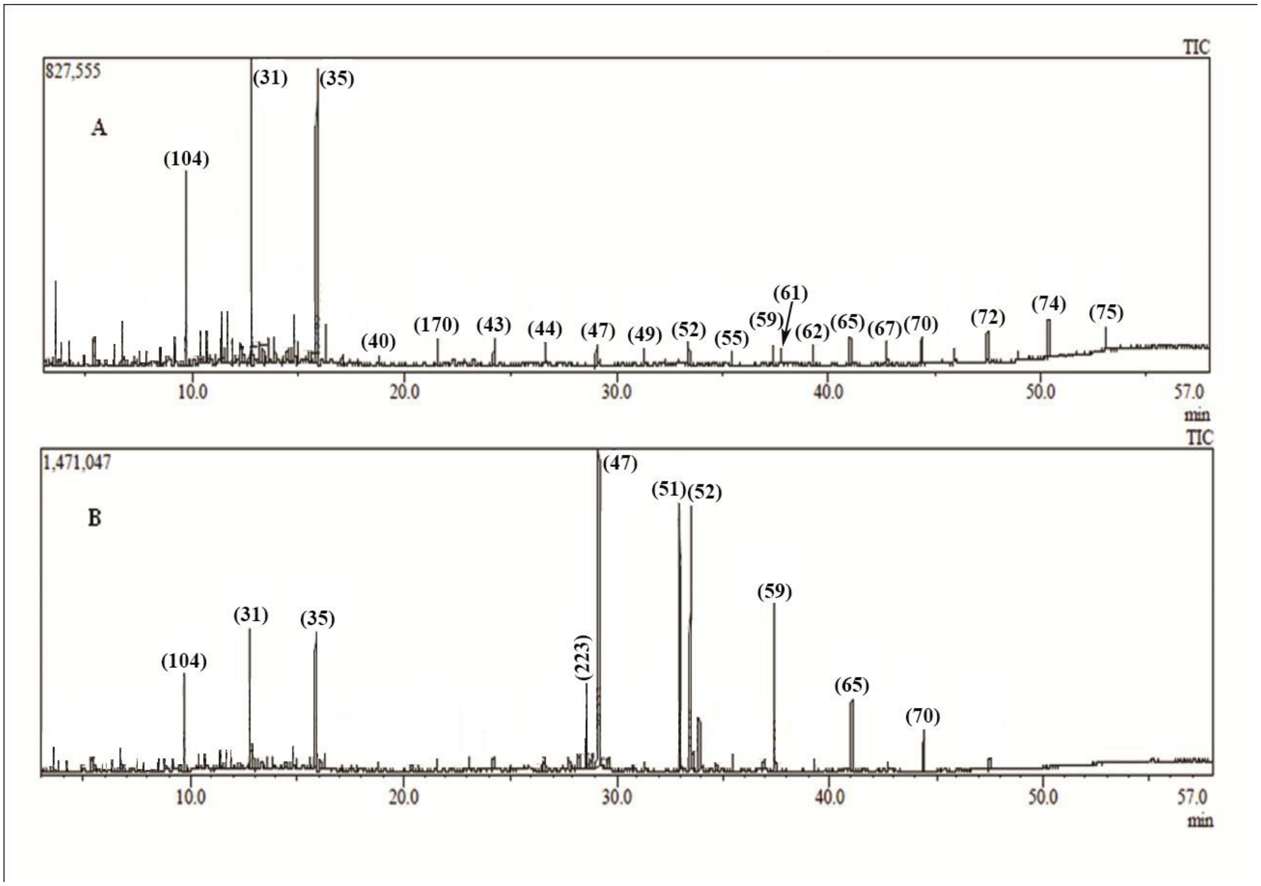

Figure S1. GC-chromatogram of the essential oil of *Rosa banksiae* var. *banksiae* Ait. (A) Aerial parts (RBW-A), (B) Flowers (RBW-F).

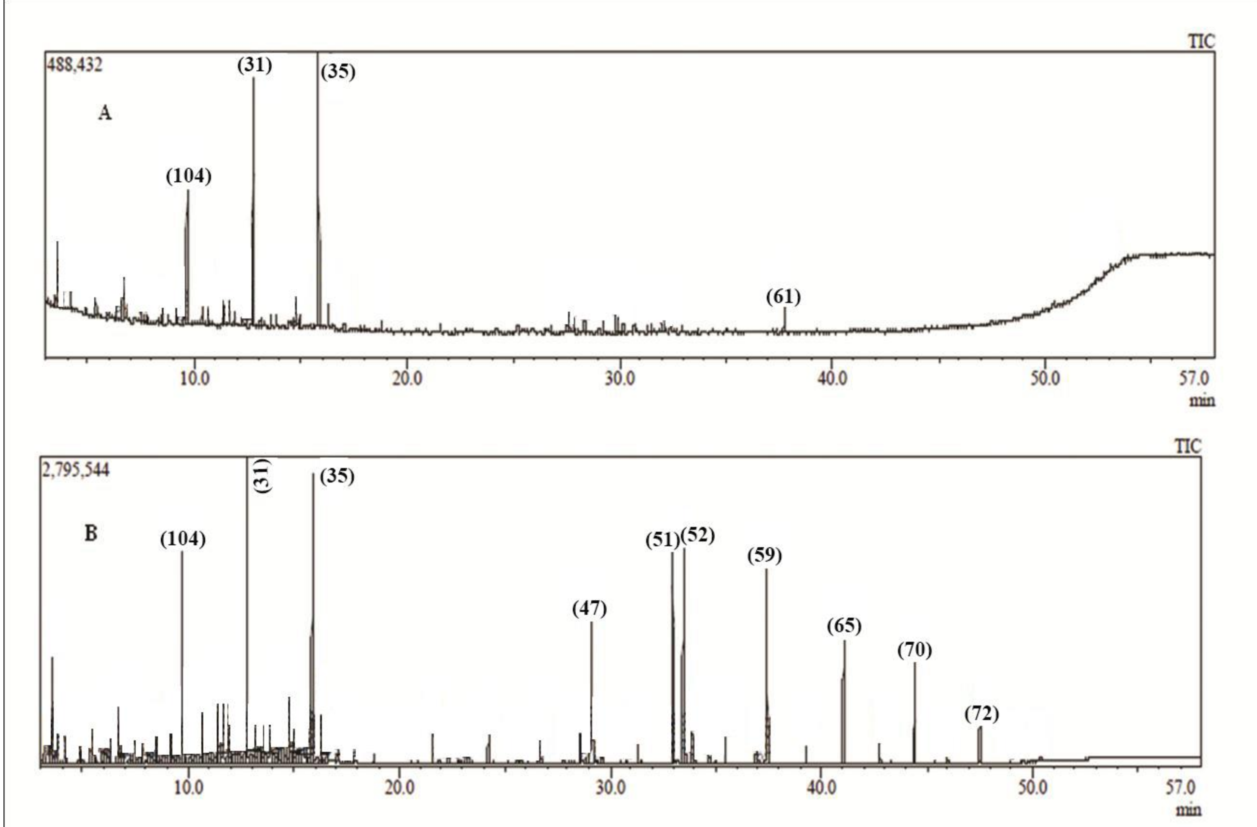

Figure S2. GC-chromatogram of the essential oil of *Rosa polyantha* Thunb. orange fairy (A) Aerial parts (RPO-A), (B) Flowers (RPO-F).

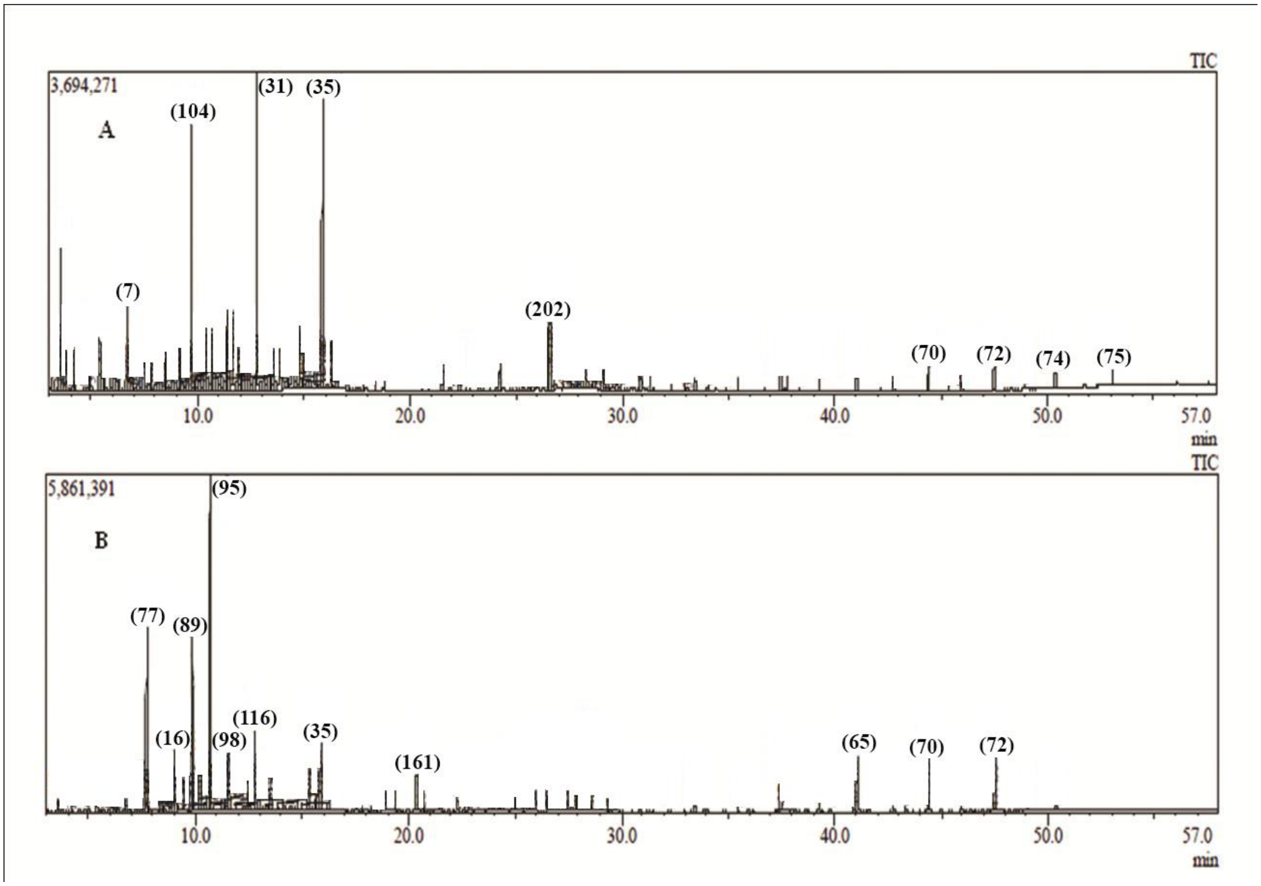

Figure S3. GC-chromatogram of the essential oil of *Rosa polyantha*Thunb. white fairy (A) Aerial parts (RPW-A), (B) Flowers (RPW-F).
